# GWAS analysis reveals the genetic basis of blast resistance associated with heading date in rice

**DOI:** 10.3389/fpls.2024.1412614

**Published:** 2024-05-21

**Authors:** Seung Young Lee, Gileung Lee, Jiheon Han, Su-Kyung Ha, Chang-Min Lee, Kyeongmin Kang, Mina Jin, Jung-Pil Suh, Ji-Ung Jeung, Youngjun Mo, Hyun-Sook Lee

**Affiliations:** ^1^ Crop Breeding Division, National Institute of Crop Science, Rural Development Administration, Wanju, Republic of Korea; ^2^ Department of Crop Science and Biotechnology, Jeonbuk National University, Jeonju, Republic of Korea; ^3^ Department of Southern Area Crop Science, National Institute of Crop Science, Rural Development Administration, Miryang, Republic of Korea; ^4^ Institute of Agricultural Science and Technology, Jeonbuk National University, Jeonju, Republic of Korea

**Keywords:** GWAS, rice blast, heading date, haplotype analysis, rice breeding

## Abstract

Rice blast is a destructive fungal disease affecting rice plants at various growth stages, significantly threatening global yield stability. Development of resistant rice cultivars stands as a practical means of disease control. Generally, association mapping with a diversity panel powerfully identifies new alleles controlling trait of interest. On the other hand, utilization of a breeding panel has its advantage that can be directly applied in a breeding program. In this study, we conducted a genome-wide association study (GWAS) for blast resistance using 296 commercial rice cultivars with low population structure but large phenotypic diversity. We attempt to answer the genetic basis behind rice blast resistance among early maturing cultivars by subdividing the population based on its *Heading date 1* (*Hd1*) functionality. Subpopulation-specific GWAS using the mixed linear model (MLM) based on blast nursery screening conducted in three years revealed a total of 26 significant signals, including three nucleotide-binding site leucine-rich repeat (NBS-LRR) genes (*Os06g0286500*, *Os06g0286700*, and *Os06g0287500*) located at *Piz* locus on chromosome 6, and one at the *Pi-ta* locus (*Os12g0281300*) on chromosome 12. Haplotype analysis revealed blast resistance associated with *Piz* locus was exclusively specific to Type 14 *hd1* among *japonica* rice. Our findings provide valuable insights for breeding blast resistant rice and highlight the applicability of our elite cultivar panel to detect superior alleles associated with important agronomic traits.

## 1 Introduction

Rice blast, caused by the fungal pathogen *Magnaporthe oryzae*, annually results in a global reduction of 10−30% in crop yields, leading to significant economic losses in the agricultural sector (Skamnioti and Gurr, 2009). The most economical method of controlling blast disease is through the breeding of resistant varieties. However, many blast resistance (R) genes are specific to certain pathogen strains, making resistant varieties susceptible to rapid diversification of blast strains (Hittalmani et al., 2000; Oliveira-Garcia and Valent, 2015). Therefore, numerous breeding programs are actively searching for diverse genetic resources to enhance resistance and are simultaneously involved in developing new rice varieties by introducing novel R genes.

To date, over 100 blast R genes have been identified, with 25 of these genes characterized at the molecular level (Kalia and Rathour, 2019). Identification of genes underlying quantitative trait loci (QTL) associated with blast resistance is usually conducted using a classic biparental map-based cloning strategy. This strategy is laborious and time consuming, and has limitations on fully elucidating the genomic potential, often resulting in only two alleles representing the entire population (Zhao et al., 2011). Genome-wide association study (GWAS) has been used to detect significant genetic variants associated with trait of interest (Varshney et al., 2009; Huang et al., 2012; McCouch et al., 2016). Compared to biparental mapping approach, the genetic diversity of the population in GWAS is considerably broader, enabling the effective mapping of alleles associated with rice blast resistance. For example, a GWAS analysis conducted with 366 diverse *indica* accessions examined under 16 rice blast strains identified 30 loci associated with blast resistance (Wang et al., 2014). Similarly, a GWAS with rice diversity panel 1 (RDP1) comprising diverse rice cultivars collected from 82 countries, has identified a total of 97 loci, of which 15 are co-localized with previously reported loci associated with blast resistance (Kang et al., 2016). Using the same panel, a GWAS was conducted to identify loci associated with field blast resistance in three major rice production areas of China (Zhu et al., 2016), and evaluation on blast resistance on diverse Chinese isolates led to the identification of a new *Pik* allele (Li et al., 2019). The GWAS with a diversity panel has indeed demonstrated its advantage in detecting several natural allelic variations controlling blast resistance. However, the validation of discovered QTL in the genetic background of local breeding population is necessary for their utilization in cultivar development (Begum et al., 2015; Verdeprado et al., 2018).

Here, we conducted GWAS on blast resistance in 296 Korean commercial rice cultivars with low population structure, yet exhibiting significant phenotypic variations across various agronomic traits (Lee et al., 2023). Our previous comparative observations based on maturity group have demonstrated that early maturing cultivars exhibit significant blast resistance compared to medium and medium-late maturing cultivars in Korea. Furthermore, association analysis among molecular markers linked to rice blast resistance has revealed that multiple R gene families at the *Piz* locus on chromosome 6 are strongly associated with blast resistance phenotype among Korean rice cultivars (Baek et al., 2021; Lee et al., 2022). Therefore, we implemented subpopulation-specific GWAS on blast resistance to discern the significant loci that contribute to the blast resistance of cultivars belonging to different maturity groups. This was implemented based on the functionality of *Heading date 1* (*Hd1*), which serves as a key determinant of heading date diversity among Korean rice cultivars (Mo et al., 2021). GWAS and haplotype analyses revealed significant blast resistance loci associated with specific *Hd1* allele types. Additionally, we discussed the geographic distribution of potential R gene donors and their direct application in breeding blast resistant cultivars in Korea.

## 2 Materials and methods

### 2.1 Plant materials and phenotypic evaluation

A total of 296 Korean commercial rice cultivars, comprising 264 *japonica* and 32 Tongil-type, were used in this study (**Supplementary Table 1**). To evaluate blast resistance, nursery tests were conducted at the experimental field station of National Institute of Crop Science (NICS) located in Wanju (35°84′ N, 127°05′ E) for three years (2018, 2019, and 2023). We established a 12-bed blast nursery, each bed measuring 45 m in length and 1.2 m in width. We directly sowed 2 g of seeds from each cultivar in 30 cm-long rows, with each adjacent row spaced 10 cm apart. The susceptible variety, Hopyung (spreader), was sown along the inner and outer bordering rows of each bed to facilitate an even spread of the blast disease. Blast infection occurred naturally, and fertilizers were applied at a ratio of N-P_2_O_5_-K_2_O = 21-17-17 kg/10a, with application of N at 80% as a basal dose, supplemented by 10% each at 12 and 20 days after sowing to induce an environment conducive to blast incidence. Additionally, we installed mesh shades over the nursery beds to reduce direct sunlight while maintaining adequate airflow, simulating natural field conditions favorable for blast infection. Disease scoring began 30 days after sowing when susceptible check (Hopyung) exhibited blast score of 7 based on standard evaluation system (IRRI, 2002). Within this system, scores of 1−3 indicate resistance, 4−6 as moderate resistance, and 7−9 as susceptibility. Monthly average air temperature, relative humidity, and rainfall data were obtained from the Korea Meteorological Administration (https://data.kma.go.kr) (**Supplementary Table 2**). Heading dates were measured in the paddy fields located at the same experimental station during the regular planting seasons of 2018 and 2023. The rice cultivars were sown on 09 May 2018 and 02 May 2023, and transplanted on 01 June 2018 and 26 May 2023, respectively. In each cultivar, 26 plants were transplanted into 4.5 m rows, with individual plants spaced 15 cm apart, and 30 cm spacing between adjacent rows. Days to heading (DTH) was determined by counting the number of days from sowing to heading when 40% of the plants within a plot exhibit emerging panicles.

### 2.2 DNA extraction and sequencing

Leaf tissues of 21-day-old seedlings from each rice cultivar were sampled, and genomic DNA was extracted using the modified cetyltrimethylammonium bromide (CTAB) method (Murray and Thompson, 1980). The quality and quantity of the extracted DNA were checked using ND-1000 spectrophotometer (Thermo Fisher Scientific, USA) and Quant-iT™ dsDNA assay kit (Thermo Fisher Scientific, USA). High-throughput SNP genotyping of 296 cultivars was performed using the KNU Affymetric Axiom Oryza 580K Genotyping array (Thermo Fisher Scientific) at DNA Link (Seoul, Korea).

### 2.3 Population structure and PCA

A set of 201,624 (minor allele frequency > 0.05, call rate = 95%) polymorphic SNPs were generated using TASSEL v5.2.80 (Bradbury et al., 2007). The phylogenic tree was constructed using TASSEL v5.2.80 (Bradbury et al., 2007) with default parameters, and was visualized by iTOL v6.8.1 (Letunic and Bork, 2021). The input file conversion from VCF to STRUCTURE was conducted using PGDSpider v2.1.1.5 (Lischer and Excoffier, 2012). The population structure of 296 cultivars was identified using STRUCTURE v2.3.4 (Pritchard et al., 2000). Run length was given as 10,000 burning period length followed by 10,000 Markov Chain Monte Carlo (MCMC) replications. Each ancestry kinship (K) value was run for 5 replications with K value varying from 1 to 10. The optimum K value was predicted by calculating ΔK according to the Evanno method (Evanno et al., 2005) using Structure Harvester (Earl and VonHoldt, 2012). Principal component analysis (PCA) was conducted using TASSEL v5.2.80 (Bradbury et al., 2007). The PCA plots of the first two components featured groups delineated based on the predicted K values.

### 2.4 GWAS analysis

High-quality SNPs were mapped on the Os-Nipponbare-Reference-IRGSP-1.0 pseudomolecule assembly (Kawahara et al., 2013) and annotated using SnpEff (Cingolani et al., 2012). We filtered 46,877 SNPs in the coding sequence (CDS) and used for GWAS. We implemented the mixed linear model (MLM) (Yu et al., 2006) and the fixed and random model circulating probability unification (FarmCPU) (Liu et al., 2016) within the ‘rMVP’ package (Yin et al., 2021) in R. Manhattan and quantile–quantile (Q–Q) plots were produced using the ‘CMplot’ package. NCBI gene bank database (https://www.ncbi.nlm.nih.gov/) and the Rice Annotation Project Database (RAP-DB) were used to validate the physical positions of the significant SNPs and retrieve corresponding gene annotation information (Sakai et al., 2013). Based on Bonferroni correction with effective number of independent SNPs at a 5% level of significance, we set the *p* threshold qualification as 0.05/number of independent SNP. The calculated threshold value was set as −log_10_ (*P*) = 5.9.

### 2.5 Nucleotide diversity, Tajima’s D, haplotype analysis, and geographical distribution

To measure the degree of polymorphism and selective pressure at SNP sites, we calculated nucleotide diversity (π) and Tajima’s *D* using VCFtools (Danecek et al., 2011). Nucleotide diversity and Tajima’s *D* indices were calculated for both candidate gene regions and whole genome, with sliding windows of 10 kb and 100 kb, respectively. Haplotype analysis was conducted using a dataset comprising 201,624 SNPs, wherein heterozygotes and missing alleles were excluded. SNPs located within the genomic regions of candidate genes annotated as NBS-LRR genes were extracted from the variant call format (VCF) using site filtering option of VCFtools (Danecek et al., 2011). Comparative phenotypic analysis of the haplotypes was performed using Scheffe’s *post hoc* test (*P* ≤ 0.05) using the ‘agricolae’ package (De Mendiburu and Simon, 2015). *Hd1* allele types of 296 cultivars were retrieved from our previous study (Mo et al., 2021). Extended haplotype network analysis was conducted using RiceVarMap v2.0 (Zhao et al., 2015) by inputting significant SNP IDs and setting the option to classification 3.

### 2.6 Statistical analysis

All statistical analyses and data visualization were performed using R version 4.2.1 (R Core Team, 2013). The best linear unbiased prediction (BLUP) value for each accession was obtained by fitting multi-season phenotype data into a linear mixed model using the ‘lme4’ package (Bates et al., 2014). Simple *t*-tests were conducted using the ‘stats’ package.

## 3 Results

### 3.1 SNP distribution and population structure of 296 rice cultivars

In this study, we performed high-throughput SNP genotyping on 296 commercial rice cultivars developed between 1979 and 2017 using Axiom genotyping platform (**Supplementary Table 1**). After data processing and SNP filtering, a total of 201,624 SNPs were physically mapped to the reference genome (IRGSP-1.0) across 12 chromosomes, with an average variant rate of 1.9 kb per chromosome (**Supplementary Figure 1**; **Supplementary Table 3**). The highest numbers of SNPs were mapped to chromosome 1 (27,378 SNPs), while the lowest number was observed on chromosome 9 (11,646 SNPs). The genetic properties of markers are summarized in **Supplementary Table 4**. The ratio of transition to transversion (ti/tv) SNPs was calculated as 2.5, with G/A, C/T, T/C, and A/G transitions collectively accounting for 17.5–18.3%, thus contributing to 71.5% of the total point mutation types.

**Figure 1:**
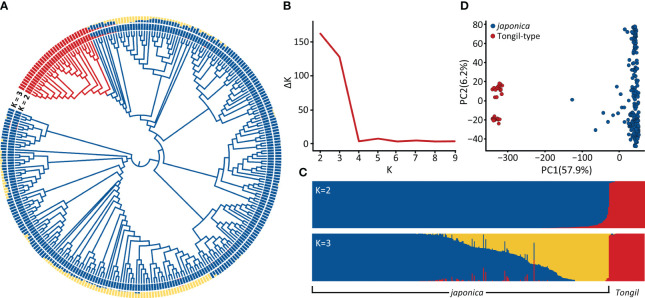
Population structure of 296 Korean rice cultivars. **(A)** Phylogenic tree constructed using high-quality 201,624 SNPs. **(B)** Plot of ΔK values ranging from 1 to 10, calculated according to the Evanno method (Evanno et al., 2005). **(C)** Genetic structure of 296 cultivars revealed by STRUCTURE with the ancestry of kinships set to 2 and 3; the proportion of genetic background of each ancestral population is indicated by vertical bar, representing *japonica* (blue), *japonica* when K=3 (yellow), and Tongil-type (red). **(D)** Principal component analysis (PCA) of the 296 cultivars.

The phylogenic tree and population structure analysis, conducted to determine the optimal K, indicated that the 296 commercial rice cultivars clustered into two subpopulations (K = 2), primarily between *japonica* (264) and Tongil-type (32) (**Figures 1A, B**). When K = 3, the *japonica* population was further subdivided into two subpopulations, while Tongil-type remained unchanged (**Figure 1C**). Similarly, the principal component analysis (PCA) showed separation into two subpopulations, elucidating a total of 64.1% genetic variation across the first two principal components (**Figure 1D**).

### 3.2 Phenotypic variations of 296 cultivars

**Figure 2:**
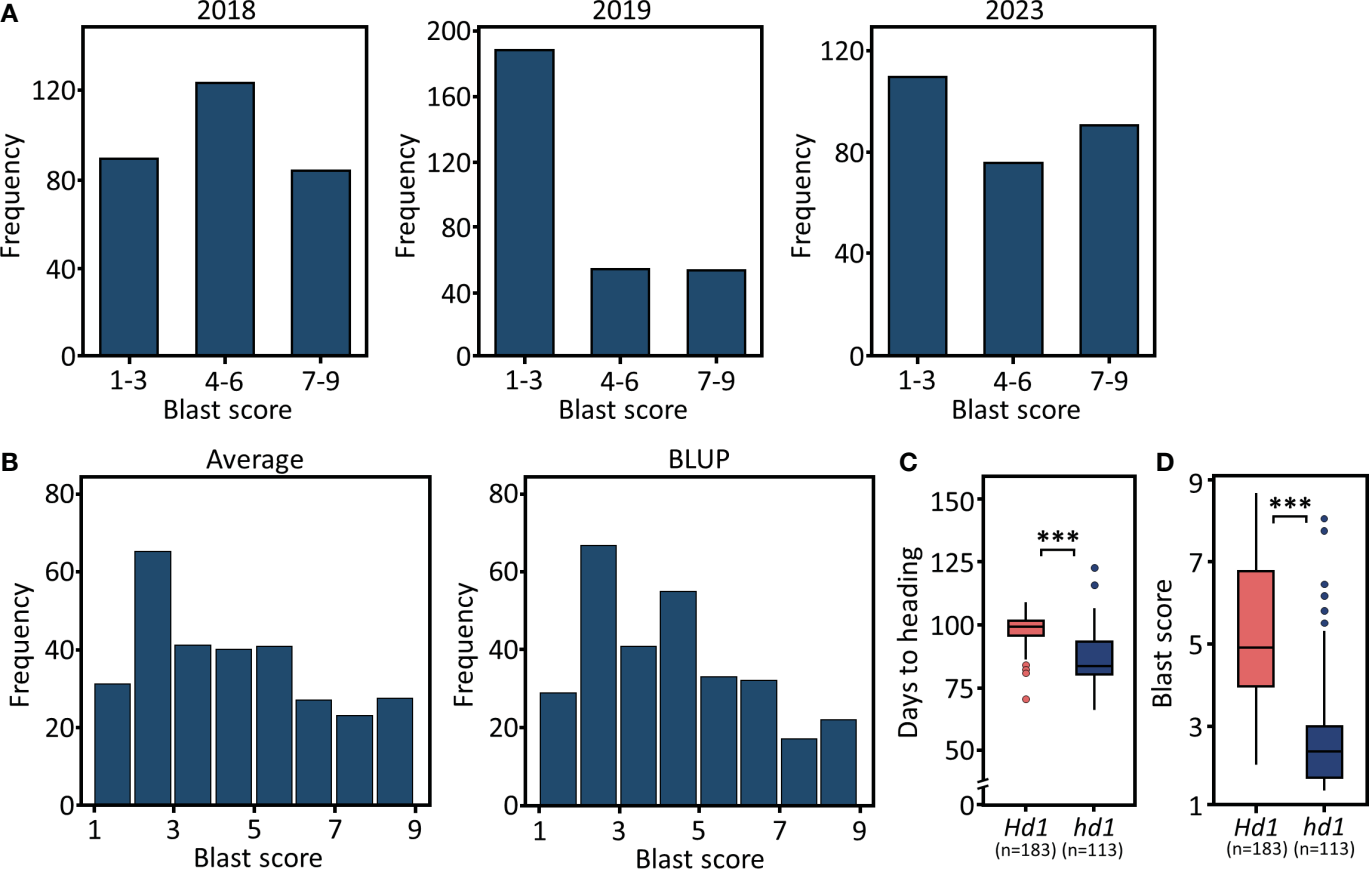
Phenotypic distribution of 296 Korean rice cultivars. **(A)** The frequency distribution of blast resistance scores across three-year trials (2019, 2020, and 2023). **(B)** Distribution of average and BLUP values for blast resistance scores. **(C)** Heading date variation based on functionality of *Hd1*. **(D)** Blast resistance score variation based on functionality of *Hd1*. ***P < 0.001.

The three-year nursery evaluation of blast resistance for 296 cultivars is presented in **Figure 2A** and **Supplementary Table 1**. The mean blast scores among three years ranged from 3.4 to 4.9. Significant correlations were observed among yearly replications, with Pearson’s correlation coefficients ranging from 0.77 to 0.79 (**Supplementary Table 5**). To enhance the precision of phenotypic predictions, we applied BLUP estimation, minimizing the sum of squared residuals and estimating random effects to bring our phenotypic data closer to a normal distribution (**Figure 2B**). Consistent with our previous observations (Mo et al., 2021; Lee et al., 2022), early maturing cultivars carrying non-functional *hd1* alleles exhibited significantly stronger blast resistance compared to mid-late maturing cultivars carrying functional *Hd1* alleles (**Figures 2C, D**).

### 3.3 Genome-wide association study

**Figure 3:**
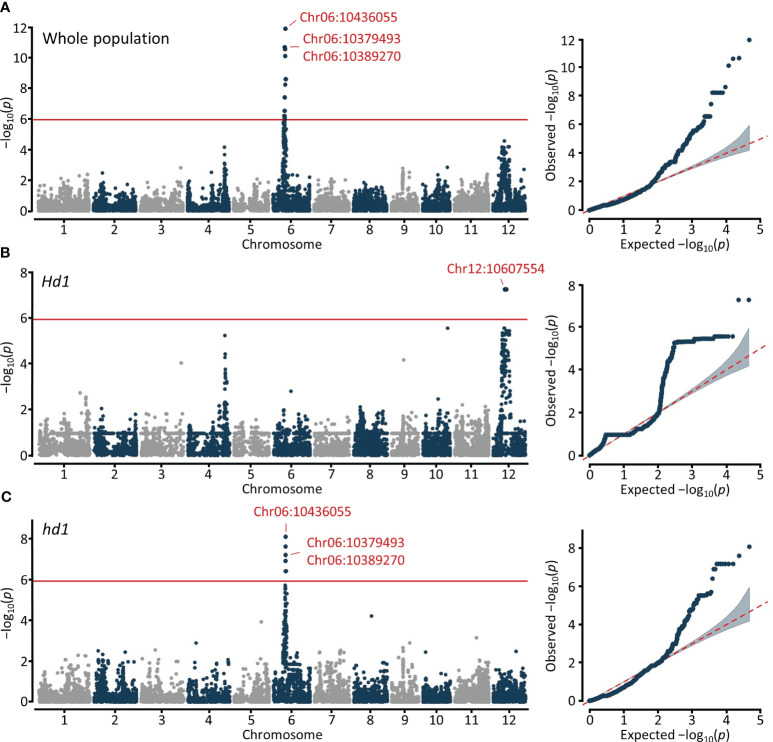
Manhattan and quantile-quantile (Q-Q) plots resulting from the GWAS for blast resistance in Korean rice cultivars using MLM. GWAS for blast resistance in **(A)** Whole population (n=296). **(B)** Functional *Hd1* group (n=183). **(C)** Nonfunctional *hd1* group (n=113). The x- and y-axis represent SNPs along each chromosome and −log_10_ (*P*) for the association, respectively. SNP positions in red indicate significant signals annotated as NBS-LRR.

For the GWAS on blast resistance, we employed 46,877 high-quality SNPs located in the coding sequences (CDS) and utilized a mixed linear model (MLM) and fixed and random model circulating probability unification (FarmCPU). To increase accuracy in predictions, we utilized BLUP values as phenotypic data for blast resistance. Based on the genome-wide significance threshold at −log_10_ (*P*) = 5.9, we identified a total of 26 and 14 signals for blast resistance across the whole (n=296), *Hd1* (n=183), and *hd1* (n=113) populations using MLM and FarmCPU, respectively (**Figure 3**; **Supplementary Figure 2**). Specifically, 12, 2, and 12 signals were detected for the whole, *Hd1*, and *hd1* populations using MLM, respectively (**Table 1**). Notably, the significant signals detected on chromosome 6 and 12 are localized within the previously reported *Piz* and *Pi-ta* loci, corresponding to nucleotide-binding site−leucine-rich repeat (NBS-LRR) proteins. The lead SNPs identified within the NBS-LRR genes were observed in both the whole and *hd1* populations, specifically located at Chr06:10379493, Chr06:10389270, and Chr06:10436055 in the *Piz* locus (**Figure 3A**). Additionally, the SNP located at Chr12:10607554 within the *Pi-ta* locus was detected only in the *Hd1* population (**Figure 3B**). These results suggest that blast resistance among early maturing cultivars is potentially associated with the *Piz* locus, while that among mid-late maturing cultivars appear to be linked with the *Pi-ta* locus.

**Table 1:** Genome-wide significant association signals for rice blast resistance using MLM.

Population	Marker name	Position	*P*-value	Gene ID	Candidate gene	Description
Whole	AX-95956321	Chr06:10124325	7.8 × 10^-11^	Os06g0284800	–	Spectrin repeat containing protein
	AX-95955430	Chr06:10184177	2.3 × 10^-11^	Os06g0285900	–	Similar to embryogenesis transmembrane protein
	AX-155658386	Chr06:10184497	2.3 × 10^-11^			
	AX-155179418	Chr06:10341729	6.0 × 10^-9^	Os06g0286310	–	Similar to Oxidoreductase-like protein
	AX-154092645	Chr06:10345821	6.0 × 10^-9^	Os06g0286351	–	Armadillo-type fold domain containing protein.
	AX-154502337	Chr06:10346650	6.0 × 10^-9^			
	AX-154313269	Chr06:10346703	6.0 × 10^-9^			
	AX-155506647	Chr06:10348330	6.0 × 10^-9^			
	AX-154528799	Chr06:10379493	6.0 × 10^-9^	Os06g0286500	*Nbs1-Pi9, Nbs1-Pi2, Nbs1-NPB*	Similar to NBS-LRR disease resistance protein homologue
	AX-273972634	Chr06:10389270	6.0 × 10^-9^	Os06g0286700	*Piz, Piz-t, Pi2, Pi9*	Similar to Piz-t
	AX-115748162	Chr06:10436055	1.3 × 10^-12^	Os06g0287500	*NBS2, Pid4*	CC-NBS-LRR protein
	AX-154765758	Chr06:10807988	2.5 × 10^-9^	Os06g0294000	–	Conserved hypothetical protein
*Hd1*	AX-115813562	Chr12:10607554	5.8 × 10^-8^	Os12g0281300	*Pi-ta*	Pi-ta protein belonging to the NBS-LRR class
	AX-123178674	Chr12:11322253	5.8 × 10^-8^	Os12g0292400	*RBCS4*	Similar to Petunia RUBSICO small subunit mRNA
*hd1*	AX-95956321	Chr06:10124325	1.3 × 10^-7^	Os06g0284800	–	Spectrin repeat containing protein
	AX-95955430	Chr06:10184177	1.3 × 10^-7^	Os06g0285900	–	Similar to embryogenesis transmembrane protein
	AX-155658386	Chr06:10184497	2.5 × 10^-8^			
	AX-155179418	Chr06:10341729	6.6 × 10^-8^	Os06g0286310	–	Similar to Oxidoreductase-like protein
	AX-154092645	Chr06:10345821	6.6 × 10^-8^	Os06g0286351	–	Armadillo-type fold domain containing protein.
	AX-154502337	Chr06:10346650	6.6 × 10^-8^			
	AX-154313269	Chr06:10346703	6.6 × 10^-8^			
	AX-155506647	Chr06:10348330	6.6 × 10^-8^			
	AX-154528799	Chr06:10379493	6.6 × 10^-8^	Os06g0286500	*Nbs1-Pi9, Nbs1-Pi2, Nbs1-NPB*	Similar to NBS-LRR disease resistance protein homologue
	AX-273972634	Chr06:10389270	6.6 × 10^-8^	Os06g0286700	*Piz, Piz-t, Pi2, Pi9*	Similar to Piz-t
	AX-115748162	Chr06:10436055	8.3 × 10^-9^	Os06g0287500	*NBS2, Pid4*	CC-NBS-LRR protein
	AX-154765758	Chr06:10807988	3.9 × 10^-7^	Os06g0294000	–	Conserved hypothetical protein

### 3.4 Nucleotide diversity, selection, and haplotype analysis

**Figure 4:**
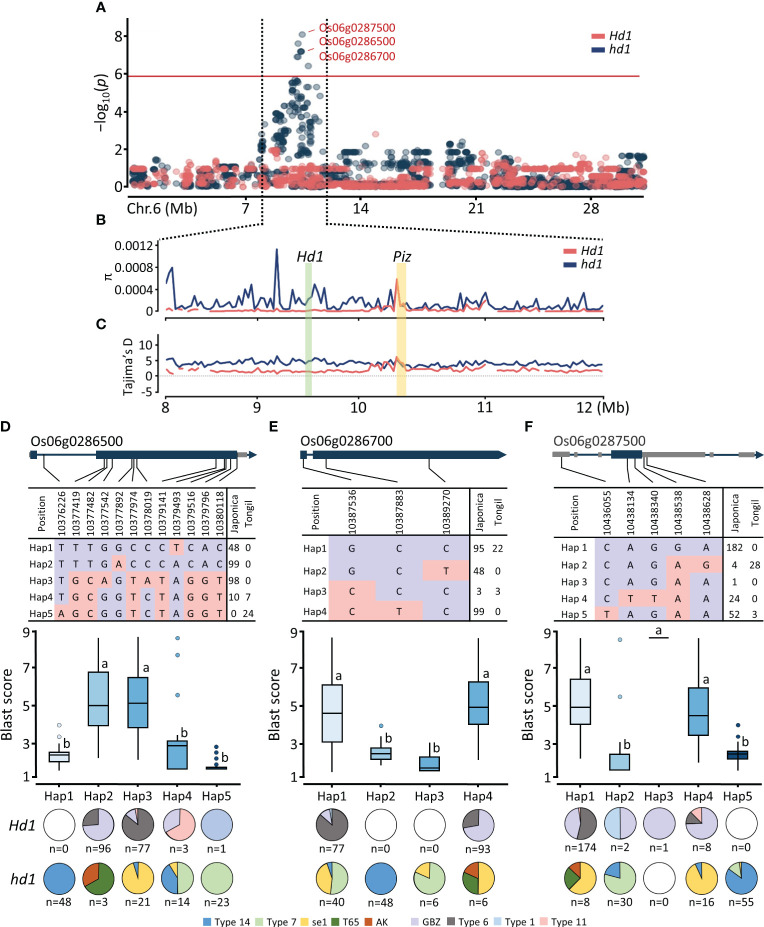
Regional Manhattan plot and haplotype analysis of *Piz* locus. **(A)** Regional Manhattan plot for the GWAS signals on chromosome 6. **(B)** Nucleotide diversity of *Hd1* and *Piz* region with 10 kb sliding window. **(C)** Tajima’s *D* of *Hd1* and *Piz* region with 10 kb sliding window. SNP variation, haplotype analysis, and distribution of *Hd1* allele types of **(D)** Os06g0286500, **(E)** Os06g0286700, and **(F)** Os06g0287500. Different lowercase letters above the box plots indicate significant differences according to Scheffe’s method for *post hoc* comparison at *P* ≤ 0.05.

Gene annotation revealed that three SNPs detected in the nonfunctional *hd1* population were associated with NBS-LRR domain proteins at *Piz* locus: *Os06g0286500*, *Os06g0286700*, and *Os06g0287500* (**Figure 4A**; **Table 1**). Since the physical distance between *Hd1* and *Piz* locus was estimated to be approximately 1 Mb according to the Nipponbare reference genome, we evaluated the degree of polymorphism and the presence of artificial selection between the functionality of *Hd1* and its relatedness with *Piz*. The nucleotide diversity of *Hd1* population was lower than that of *hd1* population within *Hd1* gene region but showed no difference in *Piz* locus (**Figure 4B**). The Tajima’s *D* values for both populations were positive, with the *Hd1* population exhibiting lower values than *hd1* (**Figure 4C**). These results suggest that the lower nucleotide diversity observed in the *Hd1* group may possibly be attributed to inbreeding depression and could have undergone domestication through balancing selection compared to the *hd1* group (Tajima, 1989).

To investigate relationship between SNP variations and distribution of *Hd1* allele types, we conducted haplotype analysis for the three NBS-LRR genes identified from the significant signals in *Piz* locus as determined by the MLM model (**Supplementary Table 6**). The allele types of *Hd1* – four functional alleles (GBZ, Type 1, Type 6, and Type 11) and five nonfunctional alleles (se1, T65, Type 7, Type 14, and AK) – in 296 Korean cultivars are based on our previous report (Mo et al., 2021). Using 12 SNPs within the gene region of *Os06g0286500*, we identified five haplotypes. Among them, the synonymous A/T SNP (Chr06:10379493) was associated with blast resistance in the *japonica* subpopulation (Hap1). Additionally, the T/A SNP (Chr06:10376226) represented a specific SNP associated with blast resistance for the Tongil-type (Hap5) (**Figure 4D**). Notably, *Os06g0286700*_Hap2 exhibited *japonica*-specific blast resistance attributed to the C/T SNP previously reported as the causal variant (Wei et al., 2021), resulting in a nonsynonymous proline-to-leucine amino acid substitution (**Figure 4E**). Furthermore, both *Os06g0286500*_Hap1 and *Os06g0286700*_Hap2 were identified only in the cultivars possessing the nonfunctional Type 14 *hd1*. As of *Os06g0287500*, we identified five major haplotypes using five SNPs in the genic region. The majority of *japonica* cultivars (*Hd1 = *174, *hd1 = *8) were categorized as Hap 1. The significant C/T SNP (Chr06:10436055), annotated as a 5’ UTR premature start codon gain variant, was associated with blast resistance only in the cultivars possessing nonfunctional *hd1* (**Figure 4F**). These findings revealed that *Piz* blast resistance is tightly linked to specific nonfunctional *hd1* allele, with the association being specific to the Type 14 *hd1* for *japonica* rice.

### 3.5 Haplotype analysis for *Pi-ta*

**Figure 5:**
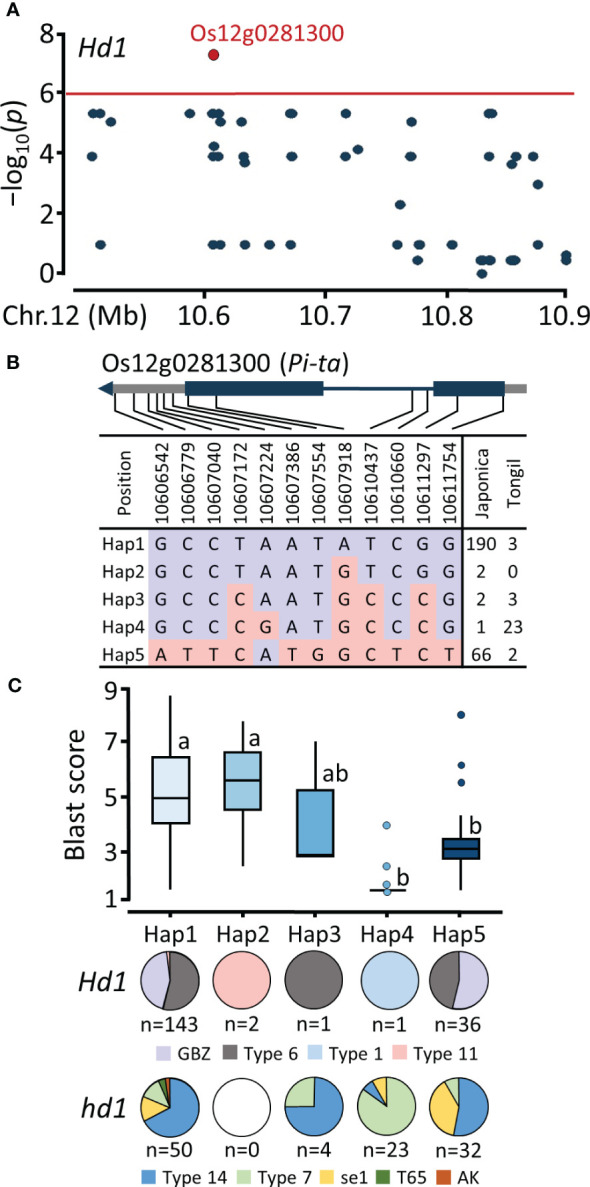
Regional Manhattan plot and haplotype analysis of *Pi-ta* locus. **(A)** Regional Manhattan plot for the candidate region on chromosome 12. **(B)** SNP variation of the Os12g0281300 gene region. **(C)** Haplotype analysis and distribution of *Hd1* allele types. Different lowercase letters above the box plots indicate significant differences according to Scheffe’s method for *post hoc* comparison at *P* ≤ 0.05.

Based on the significant signals detected in the *Pi-ta* locus through GWAS analysis using a functional *Hd1* population, we investigated the haplotype variation of *Os12g0281300*, which encodes Pi-ta protein belonging to NBS-LRR protein (**Figure 5A**). Based on 12 SNPs in the genic region, we identified five haplotypes of *Os12g0281300* (**Figure 5B**). Hap1 was predominant in the *japonica* population (n=190), comprising 143 and 50 cultivars belonging to the *Hd1* and *hd1* groups, respectively. Hap4, prevalent among Tongil-type cultivars, exhibited significant blast resistance and can be determined by a A/G SNP at the Chr12:10607224 position (**Figure 5C**). Hap5 showed significant blast resistance, consisting of both *Hd1* (n=36) and *hd1* (n=32) group, attributed to the lead T/G SNP (Chr12:10607554). This SNP resulted in a nonsynonymous serine-to-alanine amino acid substitution, causing missense variant in the coding region. These results elucidate that blast resistance associated with the *Pi-ta* locus is widely distributed among early and mid-late maturing cultivars.

### 3.6 Geographical distribution of extended haplotype variation

**Figure 6:**
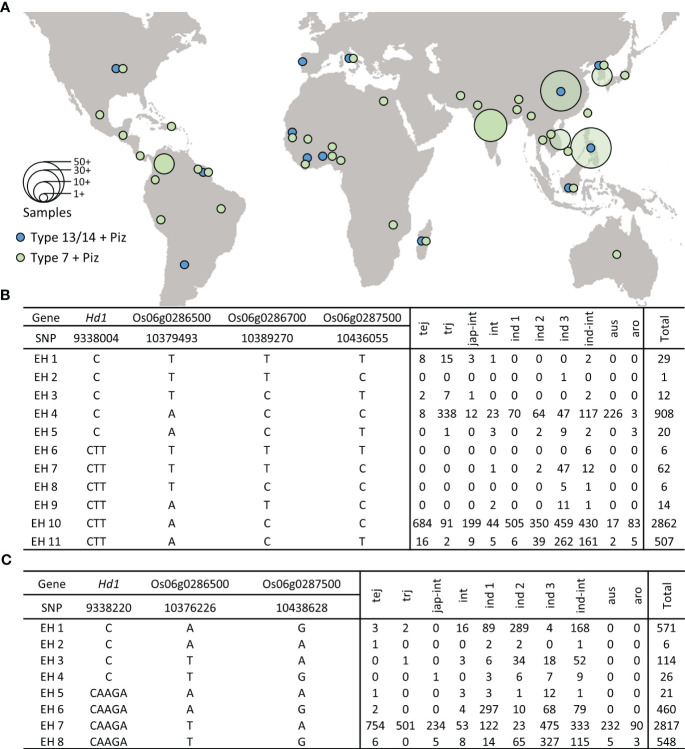
Geographical distribution and extended haplotype analysis of *Piz*. **(A)** Geographical distribution of accessions with *japonica*- and Tongil-specific *Piz* blast resistance. **(B)** Extended haplotype analysis of the SNPs significantly associated with blast resistance along with the sequence variations responsible for Type 14 *hd1*. **(C)** Extended haplotype analysis of the SNPs significantly associated with blast resistance along with the sequence variations responsible for Type 7 *hd1*. tej, Temperate *japonica*; trj, Tropical *japonica*; jap-int, *japonica*-intermediate; int, Intermediate; ind 1, *indica* I; ind 2, *indica* II; ind 3, *indica* III; ind-int, *indica*-intermediate; aus, Aus; aro, Aromatic.

Using RiceVarMap v2.0 (Zhao et al., 2015), we analyzed the extended haplotype frequency of the SNPs significantly associated with blast resistance along with the sequence variations responsible for Type 14 *hd1* (*japonica*) and Type 7 *hd1* (Tongil-type). The extended haplotype (EH) containing Type 14 *hd1* along with significant SNPs associated with blast resistance at *Piz* locus showed no clear indications of geographical distribution (**Figure 6A**). In contrast, accessions carrying Type 7 *hd1* with blast resistance were observed to be widely distributed in East and Southeast Asia, where SNPs associated with blast resistance might have originated. The 2-bp deletion in exon 2 (Yano et al., 2000), demonstrating Type 14 polymorphism (EH1–EH5) is widely distributed among tropical *japonica* and *aus* accessions. However, haplotype with tentative blast resistance is found in only a total of 29 accessions (**Figure 6B**). The 4-bp deletion in exon 2 serves as a distinguishing polymorphism of the Type 7 *hd1* allele (Mo et al., 2021). The SNPs associated with blast resistance within accessions harboring Type 7 *hd1*, were predominantly distributed among *indica* accessions (**Figure 6C**). This observation was consistent with the findings reported by (Wu et al., 2020), where the mentioned mutation was frequently identified among *indica* accessions.

## 4 Discussion

### 4.1 Utilizing elite cultivar panel as optimized breeding strategy

Crop domestication involves selective breeding of wild species, ultimately leading to the development of elite cultivars. This process is accompanied by a substantial reduction in genetic diversity, resulting in a low level of genetic variation and an increased susceptibility to genetic vulnerabilities (Meyer and Purugganan, 2013; Shi and Lai, 2015; He and Li, 2020). The narrow genetic diversity observed in the 296 cultivars examined in this study results from pre-breeding strategy, alleviating the performance gap between genetic donors and elite cultivars, aiming to facilitate the transfer of desirable trait into elite germplasm without compromising the phenotypic performance of the cultivar. For example, Saeilmi and Saeilpum are near-isogenic lines of Ilmibyeo and Ilpumbyeo, respectively. To enhance panicle blast resistance, Ilmibyeo was crossed with Hwayeongbyeo, possessing *Pb1* gene, originating from *indica* cv. Modan (Lee et al., 2015). Similarly, Saeilpum possesses *Xa3* and *Pi20*, enhancing bacterial leaf blight and leaf blast resistance derived from *indica* cv. IR24 (unpublished). Additionally, Anmi was developed by firstly transferring an introgression line derived from wild rice, *O. australiensis* (*Bph18* donor), to the *japonica* background of Jinbubyeo, then crossing with Junam, an elite *japonica* cultivar with good grain quality (Suh et al., 2014). Therefore, despite the narrow genetic diversity, a wide range of phenotypic variation has been observed among various agronomic traits of Korean rice cultivars (Mo et al., 2020; Lee et al., 2023). Although association mapping with elite cultivars may not provide the diversity required to discover novel genes, it offers practical advantages because of its direct implementation in breeding programs designed for specific local environments (Begum et al., 2015; Fujino et al., 2015; Shinada et al., 2015; Verdeprado et al., 2018; Gudi et al., 2023, Gudi et al., 2024).

### 4.2 *Pi9* associated with Type 14 *hd1* confers blast resistance

The allelic variation of *Hd1* is considered central to the heading date diversity in cultivated rice, with loss-of-function *hd1* alleles being widely utilized to develop early maturing cultivars in Korea (Takahashi et al., 2009; Mo et al., 2021). Moreover, the loss-of-function *hd1* alleles significantly contributes to the enhancement of rice adaptability in regions characterized by extended day length in high latitude areas (Fujino et al., 2019). The majority of cultivars harboring *Pi40*, an allelic variant of *Piz* (Jeung et al., 2007), exhibited early maturity and blast resistance (Cho et al., 2007; Lee et al., 2022), possibly attributed to the tight linkage between the *Hd1* and *Piz* locus on chromosome 6 (Yokoo and Fujimaki, 1971; Yokoo and Kikuchi, 1977; Fukuta et al., 2009; Telebanco-Yanoria et al., 2010; Zhou et al., 2018). In this study, we discovered the causative C/T SNP at the gene region of *Os06g0286700*, annotated as *Pi9* (Qu et al., 2006), which conferred strong blast resistance exclusively to cultivars with Type 14 *hd1* (**Figure 4E**). Consistent with our findings, studies on quantitative trait nucleotide (QTN) library revealed *Pi9* resistance allele at the same QTN site (Wei et al., 2021). In addition, the ‘T’ allele at the mentioned QTN site of *Pi9*, conferring broad spectrum resistance (Qu et al., 2006), exists in the *indica* line 75-1-127 (Liu et al., 2002; Deng et al., 2022), which was introgressed from tetraploid wild rice *O. minuta* (Amante-Bordeos et al., 1992). However, the elucidation of how the mentioned QTN site was introduced into Korean cultivars remains unclear. Type 14 *hd1* was initially reported in the *indica* rice Kasalath (Yano et al., 2000), wherein a 2-bp deletion on exon 2 was found to be abundant among tropical *japonica* and *aus* worldwide accessions, which rarely possess the *Pi9* resistance allele (**Figure 6B**). The primary donors for the *Piz* locus in Korean varieties are Japanese *japonica* varieties (Choi et al., 1989), with Jinbubyeo and Sambaegbyeo identified as secondary donors (Cho et al., 2007). Pedigree analysis of these donors suggest that *Pi9* of Korean rice varieties did not originate from *O. minuta* (Choi et al., 1989). Using *Pi9*-specific markers, Korean *japonica* varieties tested negative, whereas *Pi9* monogenic line IRBL9-W (Tsunematsu et al., 2000) exhibited positive, suggesting that the *Pi9* present in Korean *japonica* varieties may potentially belong to multi R gene family (Cho et al., 2007). The frequency of potential resistance donors of the *Pi9* allele comprises approximately 2.5% of worldwide germplasm, indicating its presence within natural variation. This suggests its probable application in enriching the genetic pool of blast resistance genes, thereby facilitating effective resistance breeding in rice.

### 4.3 Future prospects in selective breeding for blast resistant cultivars

Our prior investigation of molecular markers associated with the rice blast resistance revealed that among the multiple alleles at the *Piz* locus (*Piz*, *Pi9(t)*, *Pigm*, *Pi40*, *Piz-t/Piz-5*, *Piz-t*, *Pi2/Piz-5*, and *Pi9/Pi2*), *Pi40* exhibited the most significant association, accounting for 22.1% of the variation observed among early maturing cultivars in Korea (Lee et al., 2022). However, our results elucidated that Type 14 *hd1*-specific *Pi9* displayed a higher association, representing 55.8% of blast resistance among early maturing cultivars. Moderately resistant cultivars were with *Pi-ta* resistance, while susceptible cultivars lacked both R genes. The se1 allele type *hd1* has been widely utilized in rice breeding (Gómez-Ariza et al., 2015; Fujino et al., 2019; Leng et al., 2020), particularly in the development of early heading rice cultivars in Korea (Mo et al., 2021). However, cultivars possessing se1 type *hd1* have shown a wide range of blast scores, ranging from moderate resistance to susceptibility, prompting rice breeders to utilize early maturing cultivars with blast resistance as source for breeding. Cultivars such as Odaebyeo, Unbongbyeo, and Jinbubyeo possess Type 14 *hd1* and exhibit blast resistance. As mentioned above, they serve as primary and secondary donors of *Piz* locus and have been extensively utilized as sources for selective breeding to improve blast resistance among early maturing cultivars in Korea. Type 7 *hd1* of Tongil-type rice, an *indica*-*japonica* hybrid, exhibit relatively stronger blast resistance compared to *japonica* rice. This may be attributed to the pyramiding effect of blast-resistant genes derived from *indica* rice. Previous studies have shown that the majority of Tongil-type rice tested positive for molecular markers specific to the blast-resistant genes *Pib* and *Pigm*, which originated from *indica* rice varieties, Engkatek (Wang et al., 1999) and Gumei 4 (Deng et al., 2017), respectively.

The main objective of modern crop breeding is to achieve both high yield and quality, often resulting in a reduction of genetic diversity within the population. We observed lower nucleotide diversity across the genome in the *Hd1* population (5.3×10^-5^) compared to the *hd1* population (2.1×10^-4^). This discrepancy led to distinct patterns in the Tajima’s *D* values across the genome: while the *Hd1* population (-0.7) indicated directional selection, the *hd1* (3.5) population showed signs of balancing selection (**Supplementary Figures 3A, B**). Cultivars possessing functional *Hd1* are predominantly utilized as mid-late maturing varieties in Korea. Their prolonged vegetative stage facilitates sufficient biomass accumulation before flowering, resulting in higher yield and grain quality (Ye and Niu, 2018; Leng et al., 2020; Lee et al., 2024). This preference has driven intensified artificial selection over the past four decades, prioritizing high-yield and quality traits. The resistance gene *Pi9*, located at the *Piz* locus, has not been introduced into mid-late maturing cultivars in Korea (**Supplementary Figure 3C**). Thus, the introgression of multiple R gene hotspots from the *Piz* locus into mid-late maturing cultivars, by breaking the linkage with *Hd1*, could significantly enhance broad-spectrum blast resistance. This strategy would allow multiple R genes to recognize various effectors (Mukhtar et al., 2011; Zhang et al., 2015), providing valuable insights for the development of breeding blast resistant rice cultivars.

## 5 Conclusion

We conducted a genome-wide association study to elucidate the genetics underlying blast resistance associated with heading date among commercial rice cultivars in Korea. Through this analysis, we identified candidate genes associated with blast resistance, which were found to be specific to cultivars carrying certain allele types of *Hd1*. SNPs identified in our study provide useful resources for further gene validation and marker-assisted selection. Our study also demonstrates future insights on utilizing low-structured local commercial populations for detecting superior alleles regulating agronomic traits that can be directly adopted in breeding programs.

## Data availability statement

The datasets presented in this study can be found in online repositories. The names of the repository/repositories and accession number(s) can be found in the article/**Supplementary Material**.

## Author contributions

SYL: Conceptualization, Formal analysis, Investigation, Software, Validation, Visualization, Writing – original draft, Writing – review & editing. GL: Investigation, Writing – original draft. JH: Investigation, Writing – original draft. S-KH: Investigation, Writing – original draft. C-ML: Investigation, Writing – original draft. KK: Investigation, Writing – original draft. MJ: Investigation, Writing – original draft. J-PS: Writing – review & editing. J-UJ: Writing – review & editing. YM: Writing – review & editing. H-SL: Conceptualization, Investigation, Writing – original draft, Writing – review & editing.
