# Supplementary material for: GWAS analysis reveals the genetic basis of blast resistance associated with heading date in rice
Source: Front Plant Sci. 2024 May 21;15:1412614. doi: 10.3389/fpls.2024.1412614 (PMC11148375; doi:10.3389/fpls.2024.1412614)
Supplement: Supplementary file 1 [file DataSheet_1.docx]

Supplementary Material

# Supplementary Figures


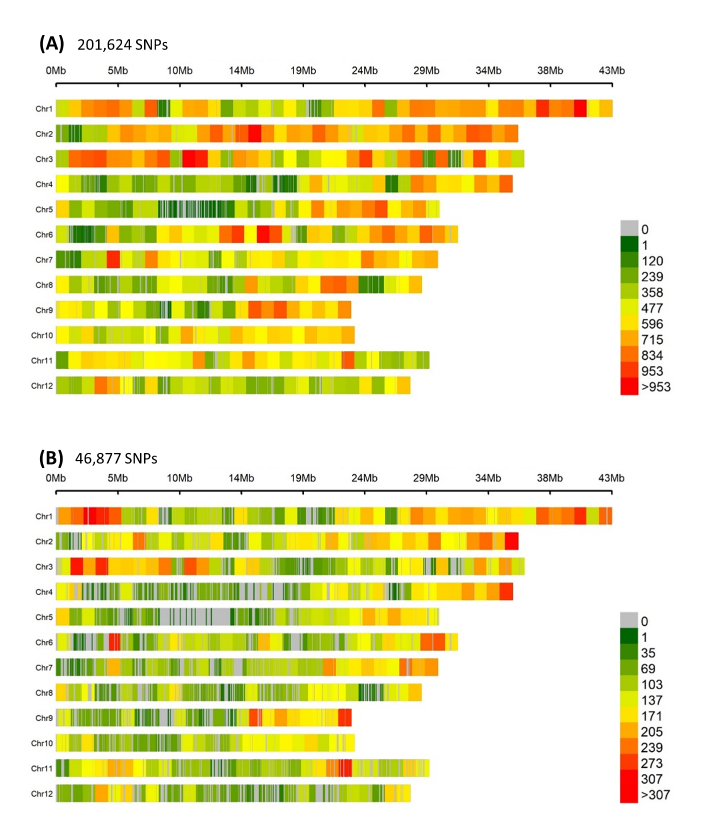


**Supplementary Figure 1.** Chromosome-wise SNP density plot representing the number of SNPs within a 1 Mb window size using **(A)** 201,624 SNPs. **(B)** 46,877 SNPs.


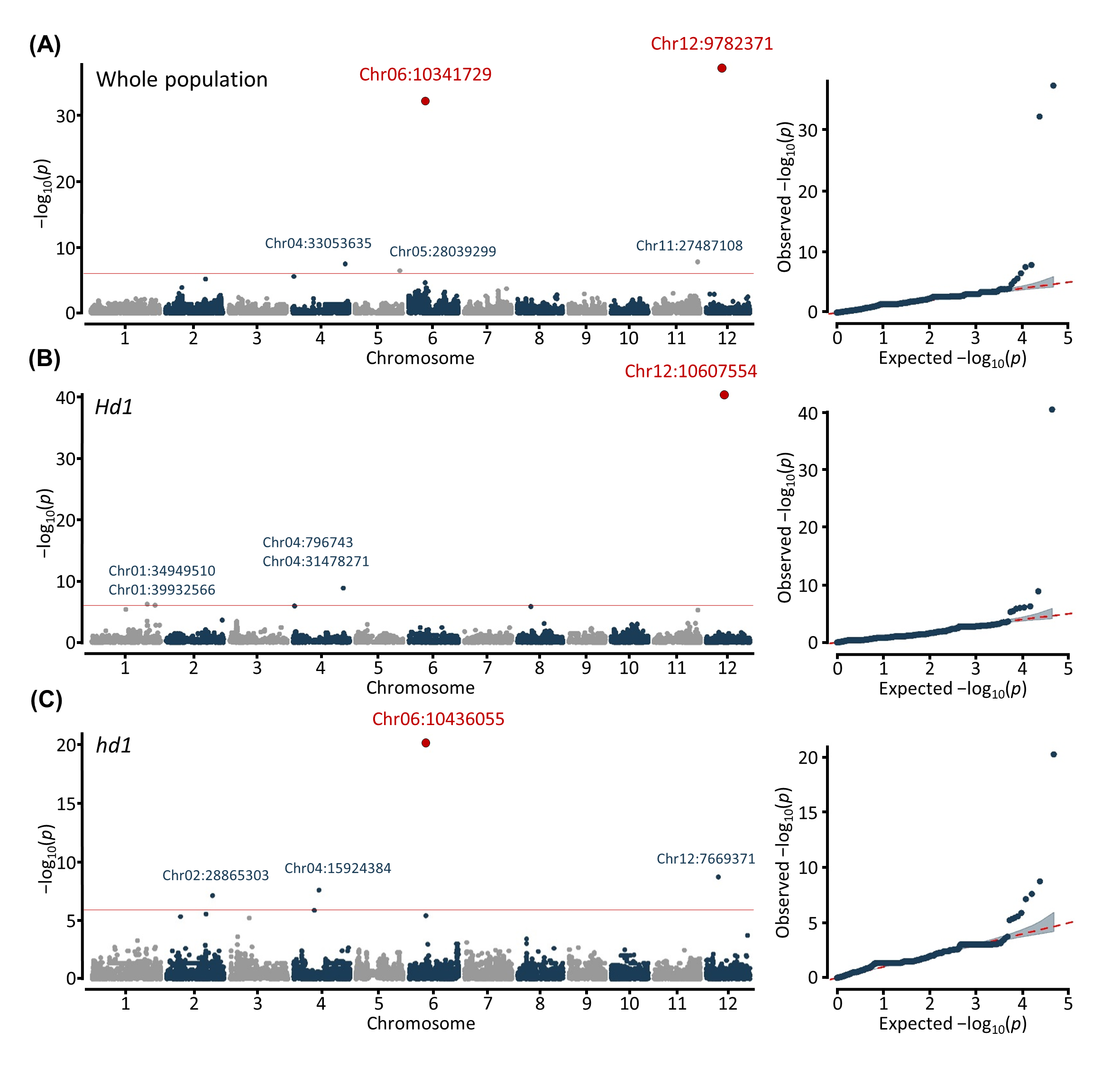


**Supplementary Figure 2.** Manhattan and quantile-quantile (Q-Q) plots resulting from the GWAS for blast resistance in rice using FarmCPU. GWAS for blast resistance in **(A)** Whole population. **(B)** Functional *Hd1* group. **(C)** Nonfunctional *hd1* group. The x- and y-axis represent SNPs along each chromosome and -log_10_ (*P*) for the association, respectively.


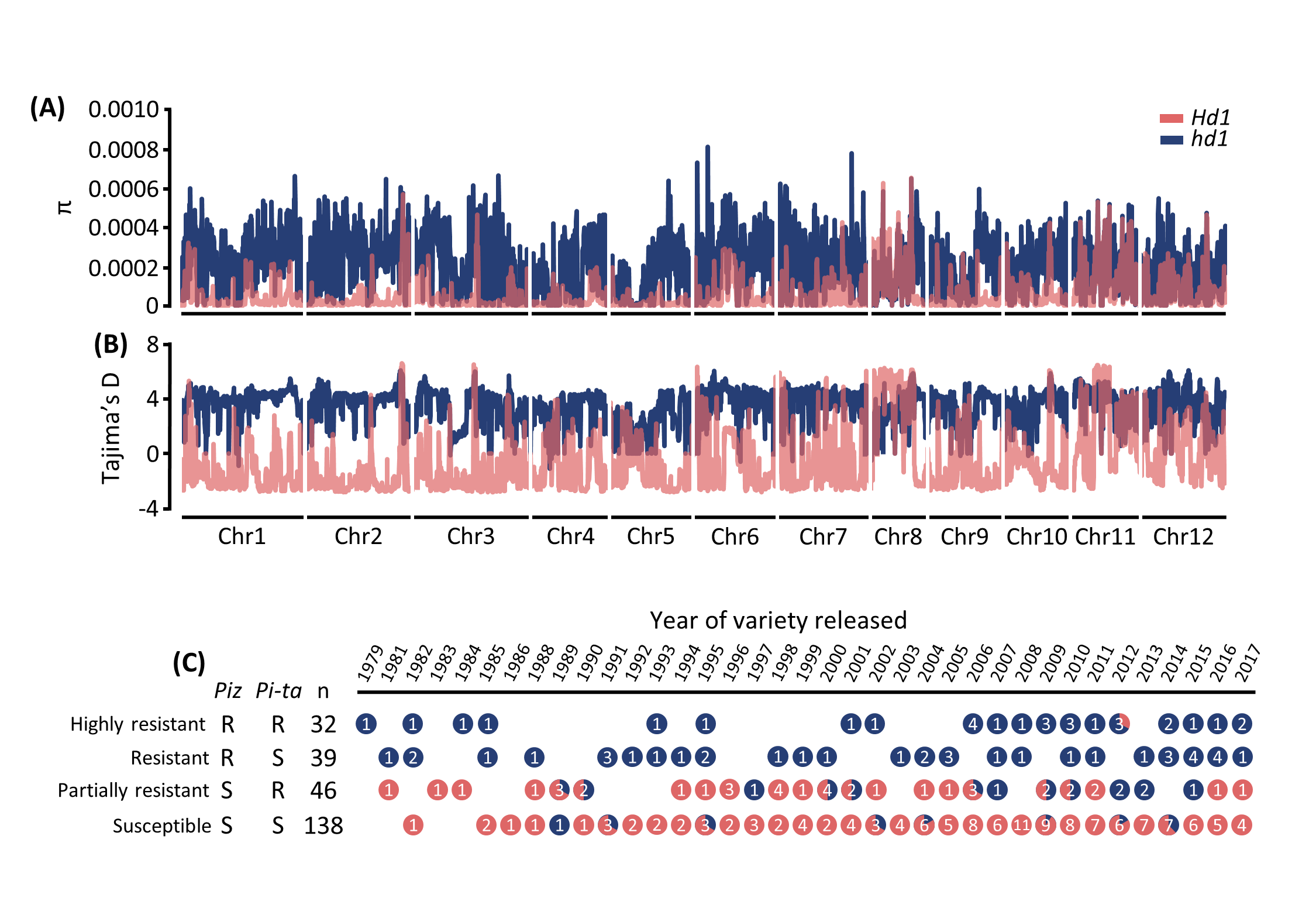


**Supplementary Figure 3.** Nucleotide diversity, Tajima’s *D*, and year of variety released. **(A)** Nucleotide diversity of whole genome with 100 kb sliding window. **(B)** Tajima’s D of whole genome with 100 kb sliding window. **(C)** Distribution of four resistant-susceptible types in cultivars released in difference breeding years. Blue and red circles indicate nonfunctional and functional *Hd1* alleles, respectively.
